# Supplementary material for: Biophysical mapping of TREM2-ligand interactions reveals shared surfaces for engagement of multiple Alzheimer’s disease ligands
Source: Res Sq. 2024 Sep 12:rs.3.rs-4850141. Preprint. [Version 1] doi: 10.21203/rs.3.rs-4850141/v1 (PMC11419269; doi:10.21203/rs.3.rs-4850141/v1)
Supplement: Supplement 1 [file NIHPPrs4850141v1-supplement-1.pdf]

Supplemental data.

Table S1. Binding sites on TREM2 basic site and CDR2 regions for IL-34 helices predicted by hydropathy mapping to have at least 75% percent match and a degree of complementary hydropathy (DCH) of at least 0.5.

Region (Residues)		Forward				Reverse			
IL-34	TREM2	Match	DCH	Start	End	Match	DCH	Start	End
Helix 2 (67-82)	Basic site and CDR2 (62-78)	75.00%	0.533	60	75	75.00%	0.545	60	75
	CDR2 and Basic site (69-78)					75.00%	0.54	71	86
	Basic site (112-114)					75.00%	0.616	112	127
Helix 5 (139-151)	Basic site (47-50)	76.92%	0.503	49	61				
	Basic site (62-68)					76.92%	0.539	53	65
	CDR2 and Basic site (69-78)	76.92%	0.538	70	82				
	Basic site (112-114)					84.62%	0.573	114	126
	Other					84.62%	0.568	117	129
Helix6 (160-181)	Basic site and CDR2 (47-75)					77.27%	0.521	49	70
	Basic site (112-114)	77.27%	0.552	100	121				

Table S2. Potential binding regions on IL-34 for TREM2 predicted by hydropathy mapping to have at least 75% percent match and a degree of complementary (DCH) hydropathy of at least 0.5. Note: Rows with text colored in red represent the top predicted binding site(s) between a pair of regions.

Region (Residues)		Forward				Reverse			
TREM2	IL-34	Match	DCH	Start	End	Match	DCH	Start	End
Basic 1 (47-50)	Negative Electrostatic Surface Potential	75.00%	0.664	40	43	75.00%	0.511	37	40
						75.00%	0.528	64	67
						75.00%	0.550	83	86

	75.00%	0.519	92	95	75.00%	0.608	90	93
	100.00%	0.658	96	99	100.00%	0.661	93	96
					75.00%	0.531	97	100
	75.00%	0.542	105	108	75.00%	0.542	105	108
	75.00%	0.606	109	112	75.00%	0.675	106	109
	75.00%	0.531	119	122	75.00%	0.531	119	122
					75.00%	0.608	122	125
					75.00%	0.586	123	126
	100.00%	0.733	126	129	75.00%	0.531	127	130
	75.00%	0.608	133	136	75.00%	0.617	136	139
	75.00%	0.542	135	138	75.00%	0.617	142	145
					75.00%	0.600	143	146
	100.00%	0.658	145	148	100.00%	0.647	146	149
	100.00%	0.667	148	151	75.00%	0.517	149	152
	100.00%	0.681	171	174	100.00%	0.669	172	175
	100.00%	0.625	174	177	100.00%	0.539	177	180
	75.00%	0.603	179	182	75.00%	0.531	183	186
	75.00%	0.603	185	188	75.00%	0.547	191	194
Positive Electrostatic Surface Potential	75.00%	0.519	57	60	75.00%	0.528	51	54
	100.00%	0.753	71	74	75.00%	0.561	69	72
	100.00%	0.697	74	77	100.00%	0.686	72	75
	75.00%	0.636	77	80	75.00%	0.553	75	78
	75.00%	0.531	155	158	100.00%	0.744	156	159
	75.00%	0.650	158	161	75.00%	0.567	161	164
	75.00%	0.664	164	167	75.00%	0.600	166	169
	100.00%	0.697	168	171	100.00%	0.681	169	172
Other	75.00%	0.519	23	26	75.00%	0.539	24	27
	75.00%	0.542	209	212	75.00%	0.525	205	208
					75.00%	0.622	230	233

		75.00%	0.556	232	235	75.00%	0.503	233	236
						75.00%	0.578	238	241
Basic 2 (62-68)	Negative Electrostatic Surface Potential	85.71%	0.667	90	96				
		85.71%	0.644	105	111				
		85.71%	0.594	122	128				
		85.71%	0.644	142	148				
	Positive Electrostatic Surface Potential	85.71%	0.684	159	165	85.71%	0.678	74	80
		85.71%	0.627	165	171	85.71%	0.586	162	168
Basic 3 (76-78)	Negative Electrostatic Surface Potential	100.00%	0.819	95	97	100.00%	0.819	95	97
		100.00%	0.804	108	110	100.00%	0.804	108	110
		100.00%	0.789	125	127	100.00%	0.789	125	127
	Positive Electrostatic Surface Potential	100.00%	0.715	163	165	100.00%	0.715	163	165
Basic 4 (112-114)	Negative Electrostatic Surface Potential	100.00%	0.641	64	66	100.00%	0.641	64	66
		100.00%	0.641	83	85	100.00%	0.641	83	85
		100.00%	0.619	93	95	100.00%	0.619	93	95
		100.00%	0.615	97	99	100.00%	0.615	97	99
		100.00%	0.526	99	101	100.00%	0.526	99	101
		100.00%	0.715	127	129	100.00%	0.715	127	129
		100.00%	0.730	136	138	100.00%	0.730	136	138
		100.00%	0.600	146	148	100.00%	0.600	146	148
		100.00%	0.626	149	151	100.00%	0.626	149	151
		100.00%	0.630	172	174	100.00%	0.630	172	174
		100.00%	0.570	175	177	100.00%	0.570	175	177
		100.00%	0.504	177	179	100.00%	0.504	177	179
		100.00%	0.667	191	193	100.00%	0.667	191	193
	Positive Electrostatic	100.00%	0.563	35	37	100.00%	0.563	35	37
		100.00%	0.619	58	60	100.00%	0.619	58	60

	Surface Potential	100.00%	0.726	72	74	100.00%	0.726	72	74
		100.00%	0.641	75	77	100.00%	0.641	75	77
		100.00%	0.715	156	158	100.00%	0.715	156	158
		100.00%	0.715	169	171	100.00%	0.715	169	171
	Other	100.00%	0.630	24	26	100.00%	0.630	24	26
		100.00%	0.678	233	235	100.00%	0.678	233	235
CDR1 (39-46)	Negative Electrostatic Surface Potential	75.00%	0.586	90	97	75.00%	0.542	93	100
		75.00%	0.535	92	99	75.00%	0.503	95	102
		75.00%	0.614	122	129				
		75.00%	0.567	142	149	75.00%	0.578	142	149
		75.00%	0.519	145	152	75.00%	0.522	145	152
		75.00%	0.521	171	178	75.00%	0.521	171	178
	Positive and Negative Electrostatic Surface Potential	87.50%	0.615	168	175	87.50%	0.615	168	175
	Positive Electrostatic Surface Potential	75.00%	0.585	71	78	75.00%	0.585	71	78
		75.00%	0.513	158	165				
		75.00%	0.554	164	171				
		75.00%	0.589	165	172	75.00%	0.600	165	172
CDR2 (69-75)	Negative Electrostatic Surface Potential	85.71%	0.632	86	92	100.00%	0.667	88	94
		85.71%	0.541	89	95	85.71%	0.565	103	109
		85.71%	0.565	115	121	85.71%	0.538	114	120
		100.00%	0.638	118	124	100.00%	0.689	117	123
		85.71%	0.514	132	138	85.71%	0.557	120	126
		85.71%	0.560	141	147	100.00%	0.624	131	137
		85.71%	0.503	176	182	85.71%	0.541	140	146
	Positive and Negative Electrostatic Surface Potential	85.71%	0.602	79	85				
		85.71%	0.606	28	34	85.71%	0.562	27	33

	Positive Electrostatic Surface Potential					85.71%	0.571	30	36
		85.71%	0.641	44	50	85.71%	0.622	43	49
		85.71%	0.597	47	53	85.71%	0.635	46	52
		100.00%	0.663	50	56	100.00%	0.625	49	55
		85.71%	0.632	73	79	85.71%	0.605	75	81
		100.00%	0.705	76	82	100.00%	0.667	78	84
		85.71%	0.594	155	161	85.71%	0.594	154	160
	Other	100.00%	0.565	25	31	85.71%	0.511	197	203
		85.71%	0.541	192	198	85.71%	0.535	200	206
		85.71%	0.540	201	207	85.71%	0.508	210	216
		85.71%	0.525	211	217	85.71%	0.522	225	231
		85.71%	0.598	229	235	100.00%	0.633	228	234
CDR3 (88-91)	Negative Electrostatic Surface Potential	100.00%	0.5	93	96	75.00%	0.514	40	43
		75.00%	0.553	105	108	75.00%	0.514	82	85
		75.00%	0.525	106	109	75.00%	0.553	105	108
		75.00%	0.542	119	122	75.00%	0.542	119	122
		75.00%	0.542	127	130	100.00%	0.572	126	129
						75.00%	0.553	135	138
						100.00%	0.506	148	151
		100.00%	0.508	172	175	100.00%	0.519	171	174
						75.00%	0.506	190	193
	Positive Electrostatic Surface Potential	100.00%	0.525	72	75	100.00%	0.592	71	74
						100.00%	0.536	74	77
		75.00%	0.503	80	83	75.00%	0.503	80	83
						75.00%	0.542	155	158
		100.00%	0.583	156	159	75.00%	0.5	158	161
						75.00%	0.514	164	167
		100.00%	0.519	169	172	100.00%	0.536	168	171
	Other	75.00%	0.528	230	233	75.00%	0.528	230	233

		75.00%	0.514	233	236	
